# Primary ileal myeloid sarcoma presenting with bowel obstruction: a case report

**DOI:** 10.1186/s40792-024-02030-5

**Published:** 2024-10-04

**Authors:** Hitoshi Minagi, Nobuhiko Kanaya, Yoshitaka Kondo, Yoshihiko Kakiuchi, Shinji Kuroda, Ryohei Shoji, Hajime Kashima, Yuki Matsumi, Satoru Kikuchi, Kunitoshi Shigeyasu, Fuminori Teraishi, Shunsuke Kagawa, Toshiyoshi Fujiwara

**Affiliations:** Department of Gastroenterological Surgery, Dentistry and Pharmaceutical Sciences, Okayama University Graduate School of Medicine, 2-5-1 Shikata-Cho, Kita-Ku, Okayama, 700-8558 Japan

**Keywords:** Myeloid sarcoma, Chloroma, Granulocytic sarcoma, Bowel obstruction, Abdominal pain

## Abstract

**Background:**

Myeloid sarcoma (MS) is an extramedullary tumor constituted by myeloid blasts or immature myeloid cells. It frequently occurs in conjunction with acute myeloid leukemia (AML); however, it can exceptionally manifest in patients without leukemia. Here, we present a rare case of primary MS originating in the small bowel without evidence of bone marrow involvement.

**Case representation:**

A 33 year-old female with no relevant medical history was admitted to our hospital with recurrent abdominal pain. Computed tomography (CT) revealed bowel obstruction due to thickening of the ileum wall, which was suspected to be an ileal tumor. Initially, ectopic endometriosis was suspected because of abdominal pain associated with the menstrual cycle and changes observed on a follow-up CT scan. The lesion could not be detected by double-balloon endoscopy. Despite conservative treatment, the obstruction persisted, and laparoscopic partial ileal resection was performed, which revealed extensive involvement of the ileum and mesentery. Additionally, the mesentery of the resected ileum was extremely thickened. Histopathological and immunohistochemical examinations of the surgical specimen indicated ileal MS. Bone marrow aspiration after discharge was negative for cytological findings of leukemia, leading to a final diagnosis of primary ileal MS. Her postoperative course was uneventful, and she is currently undergoing systemic chemotherapy tailored to AML at another hospital.

**Conclusions:**

Even though MS of the small bowel is rare and may not be considered preoperatively, similar surgical treatment to that of other small bowel malignancies can ensure proper postoperative diagnosis and appropriate chemotherapy. Given the potential need for chemotherapy, ensuring surgical safety that allows for its rapid initiation is critical.

## Background

Myeloid sarcoma (MS) is an extramedullary tumor formed by myeloid blasts or immature myeloid cells [1]. It is defined by the World Health Organization as a tumor mass consisting of myeloid blasts, with or without maturation, occurring at an anatomical site other than the bone marrow [2]. It can present anywhere in the body in a variety of ways, including as a primary lesion in acute myeloid leukemia (AML) and as a relapse after AML treatment. In the largest study using a US registry, 0.8% of patients with AML were diagnosed with MS, of which 10.3% developed MS in the gastrointestinal tract. This frequency is comparable between the synchronous and isolated MS [3]. There are relatively few reported cases of MS of the small bowel and even fewer primary cases without bone marrow involvement. This case report is significant as it presents the rarity of primary ileal MS and the difficulty of differential diagnosis from other malignancies and clarifies the clinical features of the disease based on a literature review. Herein, we describe a rare case of primary ileal MS presenting with bowel obstruction.

## Case presentation

**Fig. 1:**
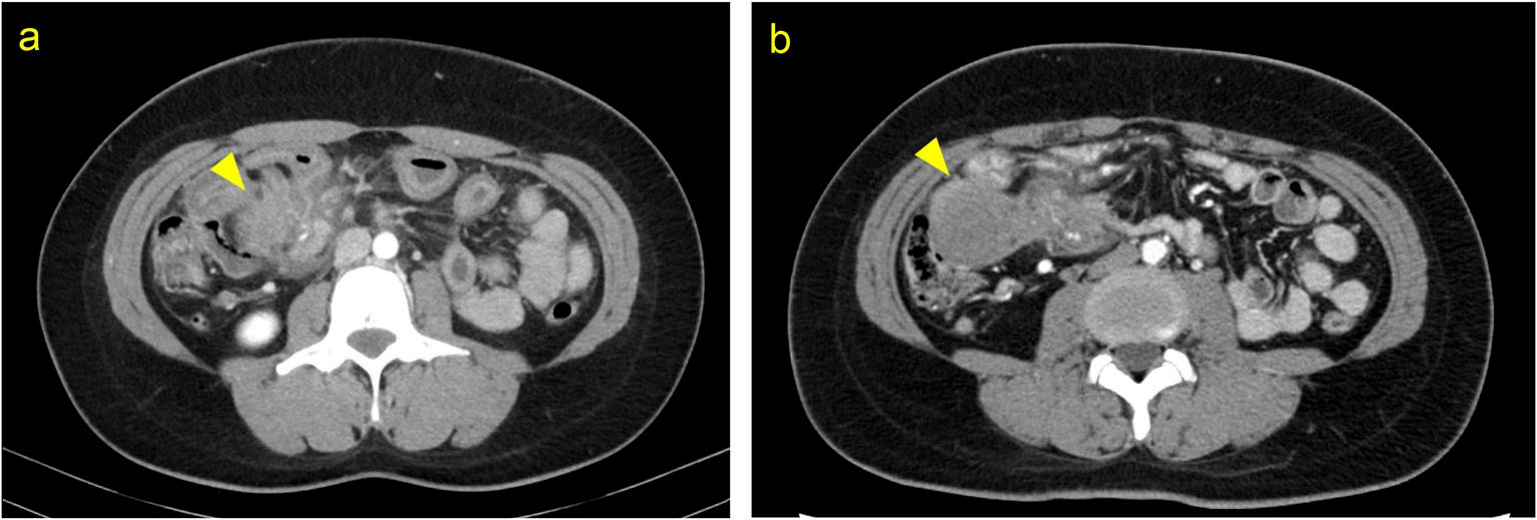
Images of contrast-enhanced computed tomography (CT). **a** CT images showing ileum with severe wall thickening and increase in surrounding fat concentration around the lesion at previous hospital (yellow triangle). **b** Follow-up CT images showing morphological changes of lumpy ileum at our hospital (yellow triangle)

A 33 year-old female with no specific medical history presented with recurrent abdominal pain over the past year. She had abdominal pain with little improvement and was referred to our hospital after a computed tomography (CT) scan showed thickening of the ileal wall and bowel obstruction. A contrast-enhanced CT scan performed at our hospital showed wall thickening with a contrast effect in the ileum, and an ileal tumor was first suspected (Fig. 1a). The lesion could not be detected by double-balloon endoscopy. Subsequently, ectopic endometriosis was suspected because of abdominal pain associated with menstruation and changes observed on a follow-up CT scan (Fig. 1b). As bowel obstruction did not improve with nonoperative trea tment, laparoscopic surgery was performed.

**Fig. 2:**
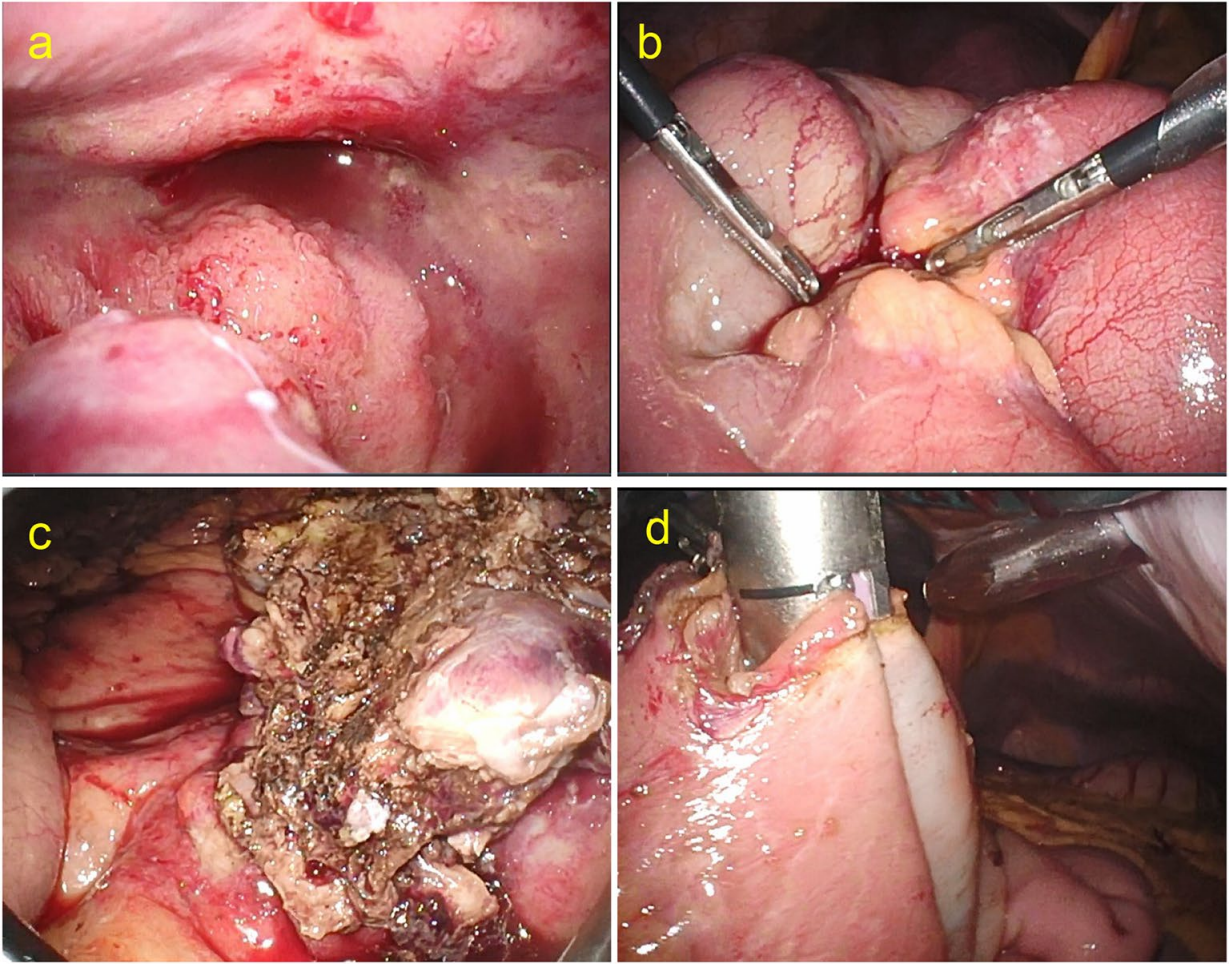
Intraoperative images for bowel obstruction. **a** Laparoscopic finding of endometriosis like diffuse small nodules in the Douglas fossa. **b** Main ileum lesion of bowel obstruction showing severe chronic inflammation. **c** Thickened mesentery at resected ileum. **d** Imaging showing functional end-to-end anastomosis (ileum to ileum) using linear cutter under the laparoscopic operation

Laparoscopic examination revealed a lumpy ileum, and the nearby mesentery was sclerotic and thickened (Fig. 2a). The lesion extended beyond the main locus into the abdominal cavity, primarily into the Douglas fossa. Laparoscopic partial ileal resection with an intracorporeal anastomosis (functional end-to-end anastomosis) was performed. The ileum was almost completely occluded by the tumor, and the nearby mesentery was extensively involved (Fig. 2b). The patient was discharged 10 days after surgery without any complications.

**Fig. 3:**
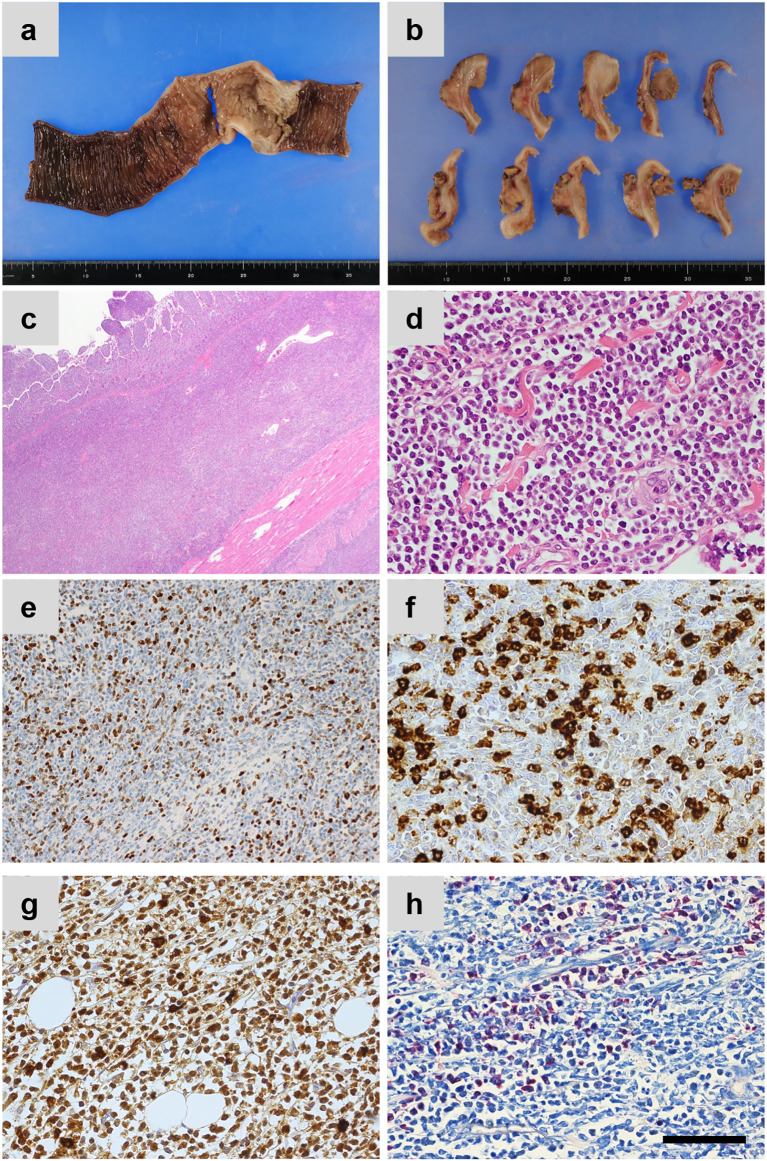
Resected specimen of primary ileal myeloid sarcoma. **a**, **b** Gross appearance. **c**, **d** Hematoxylin and eosin staining. **e**–**g** Immunofluorescence analysis of **e** Ki-67, **f** lysozyme, **g** MPO. **h** Naphthol AS-D chloroacetate esterase (NASDCA)—Giemsa staining. Scale bar: 100 μm. MPO: myeloperoxidase

Histologically, tumor cells with nuclear atypia and acidophilic cytoplasm proliferated densely across the entire ileal wall and extended into the adipose tissue of the mesentery (Fig. 3a, b).

Hematoxylin and eosin staining showed that the entire wall of the small bowel was densely occupied by medium-sized tumor cells with a high nuclear-to-cytoplasmic ratio (Fig. 3c, d).

Immunochemical staining revealed that Ki-67 was 40% positive, and the cells were positive for myeloperoxidase (MPO), lysozyme, and Naphthol AS-D chloroacetate esterase (NASDCA)Giemsa staining, leading to a diagnosis of ileal MS (Fig. 3e–h). The bone marrow aspirate obtained postoperatively was composed of normal cells with no atypical cells detected in the ileum, leading to a final diagnosis of primary ileal MS. The patient is currently admitted to another hospital for systemic chemotherapy tailored to AML.

## Discussion

We report a rare case of MS that was initially misdiagnosed as intestinal obstruction due to ectopic endometriosis. Bowel obstruction by granuloma-like lesions can be caused by Crohn’s disease, adhesions due to previous surgery, tumors, strictures related to irradiation, and ectopic endometriosis. MS can occur anywhere in the body in various ways, including as a primary lesion of AML or as a relapse after AML treatment. Nearly half of patients with MS are misdiagnosed with primary or metastatic malignancies, particularly malignant lymphomas [4]. Yamauchi et al. reported that only 53% (39) of 74 MS cases were accurately diagnosed initially. Reports indicate the difficulty of accurately diagnosis MS [4, 5]. In our case, ectopic endometriosis was suspected first because of the presence of abdominal pain associated with menstrual cycles, as well as lesions in the Douglas fossa in addition to the small bowel. MS was not included as a differential diagnosis.

Although MS is generally treated with systemic chemotherapy tailored to AML, in our case, surgery was performed to diagnose and remove bowel obstruction. Although complete resection was not achievable owing to the extent of the lesions, it might be useful to add an intraoperative pathological diagnosis of small bowel obstruction by granuloma-like lesions to determine the appropriate surgical strategy. When the intraoperative pathological diagnosis reveals a suspected hematological disease, such as MS or lymphoma, it is not necessary to aim for complete resection with a large surgical invasion; but aim for appropriate treatment without complications.

A literature search for primary MS of the small bowel between 2001 to 2023 using the keywords “primary” or “isolated” or “nonleukemic,” “myeloid sarcoma” or “chloroma” or “granulocytic sarcoma,” and “small bowel” in PubMed revealed 21 cases of primary MS of the small bowel, including our case (Table 1) [6–16]. All patients underwent surgical resection. Of these 21 patients, 16 received chemotherapy tailored to AML. One of the remaining five patients had no mention of postoperative treatment, four were treated with surgery only, and three of the four developed late-phase AML. Patients who received appropriate chemotherapy rarely developed systemic AML, and even those who developed it achieved complete remission. Few cases have been described in detail regarding residual lesions or complete resection, suggesting that it may not be a critical factor. Even with residual lesions at surgery, the disease can be managed effectively with appropriate chemotherapy.

**Table 1:** Summary of reported cases of primary MS of the small bowel

Year	Author	Age	Sex	Chief complaint	Treatment modality	Other lesions	Outcome	Dev elopment of AML or relapse of MS
2004	Mrad et al.	13	F	Abdominal mass	Surgery and chemotherapy	Unknown	27 months (alive)	None
2005	Wong et al.	36	M	Abdominal pain	Surgery and chemotherapy	Mesentery, peritoneal fluid	1 year (alive)	None
2007	Jung et al.	48	M	Abdominal discomfort	Surgery (biopsy and bypass), chemotherapy and BMT	Multiple nodules in mesentery and peritoneal	6 months (alive)	AML (4 weeks) → CR
2008	Lee et al.	45	M	Abdominal pain	Surgery and chemotherapy	Unknown	12 months (alive)	None
2008	Kitagawa et al.	33	F	Abdominal pain and vomiting	Surgery, chemotherapy and BMT	Unknown	4 years (alive)	None
2009	McKenna et al.	50	F	Abdominal pain	Surgery and chemotherapy	Unknown	2 years (alive)	None
2009	Palanivelu et al.	52	M	Abdominal distension and pain	Surgery	Unknown	14 months (alive)	None
2009	Kumar et al.	55	F	Abdominal pain and vomiting	Surgery and chemotherapy	Multiple nodules in mesentery and small bowel	Not described	Not described
2009	Ioannidis et al.	48	M	Epigastric pain, distension, vomiting	Surgery and chemotherapy	Greater omentum	6 months (alive)	None
2011	Kwan et al.	39	F	Abdominal pain, nausea, vomiting and diarrhea	Surgery, steroid therapy and chemotherapy	Unknown	2 years (alive)	None
2012	Kim et al.	49	M	Abdominal pain	Surgery	Unknown	7 months (dead)	Lung and liver masses
2013	Hotta and Kunieda	50	M	Vomiting	Surgery and chemotherapy	Unknown	36 months (alive)	None
2014	Yoldaş et al.	44	M	Abdominal pain, distension, nausea and vomiting	Surgery and chemotherapy	None (No visible residual lesions at surgery)	9 months (alive)	None
2014	Gajendra et al.	35	M	Abdominal pain	Surgery	Multiple bowel lesions (Not resected)	Not described	AML (1 month)
2016	McCusker et al.	22	F	Abdominal pain	Surgery, CHOP therapy, chemotherapy and BMT	Unknown	13 months (alive)	None
2017	Wang et al.	25	M	Abdominal distension	Surgery	Both kidney (Not resected)	10 months (alive)	Multiple intra- abdominal masses (3 months) → chemotherapy → CR
2017	Cicilet et al.	45	F	Abdominal pain and vomiting	Surgery (no description of postoperative treatment)	None (No visible residual lesions at surgery)	Not described	Not described
2018	Nemésio et al.	42	F	Abdominal pain	Surgery and chemotherapy	None (No visible residual lesions at surgery)	2 years (alive)	None
2018	He et al.	40	M	Abdominal pain	Surgery and chemotherapy	None (No visible residual lesions at surgery)	Not described	None
2020	Mizumoto et al.	54	M	Abdominal pain and vomiting	Surgery and chemotherapy	None (No visible residual lesions at surgery)	6 months, alive	None
2024	our case	33	F	Abdominal pain	Surgery and chemotherapy	Multiple intra-abdominal masses		

M: male; F: female; AML: acute myeloid leukemia; MS: myeloid sarcoma; BMT: bone marrow transplantation; CHOP: cyclophosphamide/hydroxydaunomycin/oncovirin/prednisone; CR: complete remission

## Conclusions

Even though MS of the small bowel is rare and may not be considered preoperatively, similar surgical treatment to that for sarcomas or lymphomas ensures proper postoperative diagnosis and appropriate chemotherapy, thereby minimizing the impact on patient prognosis. Given the potential need for chemotherapy in small bowel malignancies, ensuring surgical safety that allows for its rapid initiation is critical.

## Data Availability

The datasets supporting the conclusions of this study are included in this article and its additional files.

## Abbreviations

MS: Myeloid sarcoma
AML: Acute myeloid leukemia
CT: Computed tomography
TdT: Terminal deoxynucleotidyl transferase
MPO: Myeloperoxidase
FDG-PET: ^18^F-fluorodeoxyglucose-positron emission tomography
